# Human Cytomegalovirus miR-US5-2 Downregulation of GAB1 Regulates Cellular Proliferation and *UL138* Expression through Modulation of Epidermal Growth Factor Receptor Signaling Pathways

**DOI:** 10.1128/mSphere.00582-20

**Published:** 2020-08-05

**Authors:** Meaghan H. Hancock, Jennifer Mitchell, Felicia D. Goodrum, Jay A. Nelson

**Affiliations:** a Vaccine and Gene Therapy Institute, Oregon Health and Science University, Beaverton, Oregon, USA; b Department of Immunobiology, BIO5 Institute, University of Arizona, Tucson, Arizona, USA; University of North Carolina, Chapel Hill

**Keywords:** GAB1, UL138, cytomegalovirus, miRNA

## Abstract

Regulation of epidermal growth factor (EGF) receptor (EGFR) signaling is critical for the replication of human cytomegalovirus (HCMV) as well as latency and reactivation in CD34^+^ hematopoietic progenitor cells. HCMV microRNAs (miRNAs) provide a means to modulate the signaling activated by EGF through targeting components of the EGFR signaling pathways. Here, we demonstrate that HCMV miR-US5-2 directly downregulates the critical EGFR adaptor protein GAB1 that mediates activation and sustained signaling through the phosphatidylinositol 3-kinase (PI3K) and MEK/extracellular signal-regulated kinase (ERK) pathways and cellular proliferation in response to EGF. Expression of HCMV UL138 is regulated by the transcription factor early growth response gene 1 (EGR1) downstream of EGFR-induced MEK/ERK signaling. We show that by targeting GAB1 and attenuating MEK/ERK signaling, miR-US5-2 indirectly regulates EGR1 and UL138 expression, which implicates the miRNA in critical regulation of HCMV latency.

**IMPORTANCE** Human cytomegalovirus (HCMV) causes significant disease in immunocompromised individuals, including transplant patients. HCMV establishes latency in hematopoietic stem cells in the bone marrow. The mechanisms governing latency and reactivation of viral replication are complex and not fully understood. HCMV-encoded miRNAs are small regulatory RNAs that reduce protein expression. In this study, we found that the HCMV miRNA miR-US5-2 targets the epidermal growth factor receptor (EGFR) adaptor protein GAB1 which directly affects downstream cellular signaling pathways activated by EGF. Consequently, miR-US5-2 blocks the EGF-mediated proliferation of human fibroblasts. Early growth response gene 1 (EGR1) is a transcription factor activated by EGFR signaling that regulates expression of HCMV UL138. We show that miR-US5-2 regulates UL138 expression through GAB1-mediated downregulation of the signaling pathways that lead to EGR1 expression. These data suggest that miR-US5-2, through downregulation of GAB1, could play a critical role during reactivation from latency by reducing proliferation and UL138 expression.

## Abstract (precis)

Human cytomegalovirus (HCMV) causes significant disease in immunocompromised individuals, including transplant patients. HCMV establishes latency in hematopoietic stem cells in the bone marrow. The mechanisms governing latency and reactivation of viral replication are complex and not fully understood. HCMV-encoded miRNAs are small regulatory RNAs that reduce protein expression. In this study, we found that the HCMV miRNA miR-US5-2 targets the epidermal growth factor receptor (EGFR) adaptor protein GAB1 which directly affects downstream cellular signaling pathways activated by EGF. Consequently, miR-US5-2 blocks the EGF-mediated proliferation of human fibroblasts. Early growth response gene 1 (EGR1) is a transcription factor activated by EGFR signaling that regulates expression of HCMV UL138. We show that miR-US5-2 regulates UL138 expression through GAB1-mediated downregulation of the signaling pathways that lead to EGR1 expression. These data suggest that miR-US5-2, through downregulation of GAB1, could play a critical role during reactivation from latency by reducing proliferation and UL138 expression.

## INTRODUCTION

Human cytomegalovirus (HCMV) is a member of the betaherpesvirus family that successfully infects between 40% and 90% of the world’s population. HCMV has a broad host tropism with the capacity to productively infect numerous cell types, including fibroblasts and epithelial and endothelial cells as well as differentiated cells of the myeloid lineage. In contrast, undifferentiated myeloid lineage cells, such as CD14^+^ monocytes and CD34^+^ hematopoietic progenitor cells (HPCs), support latent HCMV infection, where the viral genome is maintained but no new progeny viruses are produced (1). Only a few gene products have been investigated during latency in CD34^+^ HPCs, including LUNA (2, 3), viral microRNAs (miRNAs) (4–8), US28 (9–13), and proteins from the UL133-to-UL138 locus (14–19), all of which have known functions in modulating the ability of the cells to sense and respond to extracellular signals. The ability to undergo vastly different viral gene expression programs in different cell types requires the virus to manipulate cellular signaling pathways to regulate viral and cellular gene expression.

HCMV encodes 22 mature microRNAs (miRNAs), which are small, regulatory RNAs that act as the mRNA recognition component of the multiprotein RNA-induced silencing complex (RISC) (20). miRNAs bind to regions of complementarity, normally within the 3′ untranslated region (3′ UTR) of the mRNA, through nucleotides 2 to 8 of the miRNA seed region. The miRNA/mRNA interaction results in either translational repression or mRNA degradation, ultimately resulting in reduced protein expression (21). Experimental identification and validation of HCMV miRNA targets have uncovered novel regulatory mechanisms utilized by the virus. miRNAs represent powerful tools for HCMV to regulate protein expression since they are encoded by minimal genetic material and yet have the potential to regulate expression of hundreds of cellular and viral genes (22). Their nonimmunogenic nature makes viral miRNAs ideal tools to fine-tune protein expression during latency. HCMV miRNAs are involved in immune evasion, blocking of proinflammatory cytokine production and signaling, cell survival, and virion assembly compartment formation (20) as well as in modulating cell proliferation and differentiation, viral gene expression, and signaling during latency in CD34^+^ HPCs (5, 8).

Signaling through the epidermal growth factor (EGF) receptor (EGFR) inhibits lytic viral replication (16) and has recently been identified as an important modulator of HCMV latency and reactivation (5, 14–16). EGF binding to the EGFR activates numerous downstream signaling pathways, culminating in regulation of cellular processes such as apoptosis, protein synthesis, and actin cytoskeletal rearrangement as well as activation of transcription factors responsible for proliferation and cellular differentiation (23). Given the key role of EGFR in cell homeostasis, it is not surprising that receptor expression levels, localization, and signaling are precisely regulated by RNA and DNA viruses (15, 16, 24–30). HCMV transcriptionally downregulates EGFR during lytic infection and targets EGFR for turnover (14, 16, 27) but utilizes the receptor during entry into monocytes and CD34^+^ HPCs, where EGFR signaling is critical for trafficking of the viral genome to the nucleus (31–34). Previous work demonstrated that the presence of UL138 is critical for the maintenance of EGFR cell surface expression and downstream signaling in CD34^+^ HPCs (14, 16). In turn, expression of UL138 is regulated by the transcription factor early growth response gene 1 (EGR1), whose expression is induced by EGF-mediated MEK/extracellular signal-regulated kinase (ERK) signaling (5, 14). Since UL138 acts to maintain EGFR surface levels and MEK/ERK signaling, a feed-forward loop of EGR1 expression, UL138 expression, and EGFR surface levels is established during infection.

EGFR signals are propagated using adaptor proteins that associate with the activated receptor, including the Grb2-associated binding protein (GAB) family of docking proteins that are critical for downstream signaling through both the MEK/ERK and phosphatidylinositol 3-kinase (PI3K) pathways (35). All GAB proteins contain N-terminal plextrin homology (PH) domains that can bind phosphatidylinositol lipids within membranes, several proline-rich motifs which serve as binding sites for SH3-containing proteins (such as Grb2 and the tyrosine kinase Src), and multiple tyrosine phosphorylation sites for recruiting SH2-containing and PTB-containing effectors, including the PI3K regulatory subunit p85 and SHP2, which are essential for propagating EGF-induced signals to the PI3K/AKT and MEK/ERK signaling pathways, respectively (36). The relative abundance of GAB1 is the critical factor in controlling activation of the PI3K and MEK/ERK pathways, which is especially important under conditions of low EGF or EGFR expression (37).

In the current study, we identified GAB1 as a target of HCMV miR-US5-2 and demonstrated that targeting of GAB1 results in altered EGF-mediated MEK/ERK and PI3K signaling and cellular proliferation in human fibroblasts. Through attenuating EGF-mediated signaling, miR-US5-2 interferes with expression of the HCMV protein UL138, thus highlighting the complex interplay between viral miRNAs and host and viral proteins during HCMV infection.

## RESULTS

### GAB1 is a target of HCMV miR-US5-2.

**FIG 1:**
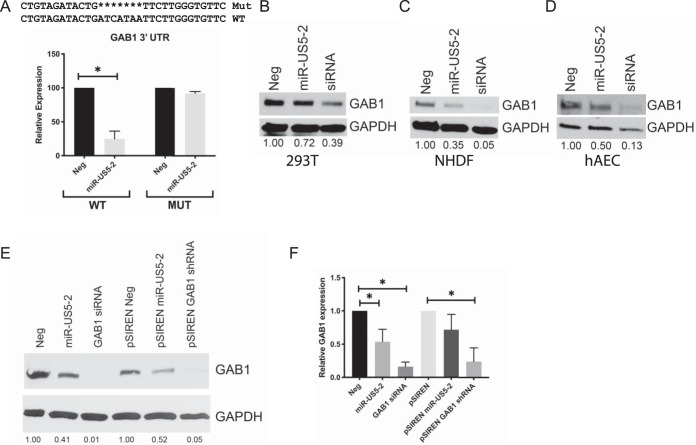
GAB1 is a target of HCMV miR-US5-2. (A) The GAB1 3′ UTR or the same 3′ UTR lacking the miR-US5-2 target site was cloned into a Dual-Luciferase reporter vector and transfected into HEK293T cells along with negative-control (Neg) miRNA or miR-US5-2 mimic. 24 h later, cells were lysed, and luciferase expression was measured. Experiments were performed in triplicate. MUT, mutant. (B to D) HEK293T cells (B), normal human dermal fibroblasts (NHDF) (C), and human aortic endothelial cells (hAEC) (D) were transfected with negative-control miRNA, miR-US5-2 mimic, or a GAB1 siRNA. 48 h later, cells were lysed and subjected to immunoblotting for GAB1 and GAPDH. GAB1 band intensity was calculated using ImageJ software and compared to GAPDH band intensity. The ratio of GAB1 band intensity to GAPDH band intensity was set to 1 for the Neg sample, and each subsequent sample ratio is presented as a multiplier of the value corresponding to the Neg time point. (E) HEK293T cells were transfected with negative-control miRNA, miR-US5-2 or a GAB1 siRNA, or expression vectors expressing the miR-US5-2 hairpin or an shRNA targeting GAB1. 48 h later, cells were lysed and immunoblotted as described for panels B to D. (F) HEK293T cells were transfected as described for panel E, and RNA was harvested 48 h later. GAB1 mRNA expression levels were determined using qRT-PCR and normalized to 18S expression levels. Experiments were performed in triplicate. Data are presented as standard errors of the means. *, *P* < 0.05 (as determined by two-tailed two-sample *t* test).

Since EGFR signaling is regulated during lytic infection and is important for latency and reactivation in CD34^+^ HPCs (5, 14–16, 31), we examined EGFR signaling pathway members as potential targets of HCMV miRNAs. Biochemical and bioinformatic (38) analyses indicated that EGFR adaptor protein GAB1 was a potential target of HCMV miR-US5-2. To determine if GAB1 expression was regulated by miR-US5-2, the wild-type (WT) GAB1 3′ UTR and a 3′ UTR containing a deletion of the putative miR-US5-2 binding site were cloned into a Dual-Luciferase reporter plasmid and transfected into HEK293T cells along with negative-control miRNA or miR-US5-2 mimic (Fig. 1A). miR-US5-2 mimic was capable of downregulating luciferase expression from the WT GAB1 3′ UTR, but not the mutated 3′ UTR, indicating that miR-US5-2 regulates the GAB1 3′ UTR through the identified site. We next tested the effect of miR-US5-2 on endogenous GAB1 protein levels in a variety of cell types. Transfection of miR-US5-2 mimic into HEK293T cells (Fig. 1B), normal human dermal fibroblasts (NHDF) (Fig. 1C), or primary human aortic endothelial cells (hAECs) (Fig. 1D) resulted in decreased GAB1 protein expression compared to negative-control transfected cells. We also cloned a 500-bp region surrounding miR-US5-2 from the HCMV genome, or that of a short hairpin RNA (shRNA) targeting GAB1, into a pSIREN expression vector. Transfection of HEK293T cells with these vectors resulted in decreased protein expression compared to negative-control transfected cells (Fig. 1E), indicating that miR-US5-2 processed from the miRNA’s native conformation is capable of downregulating GAB1. Analysis of GAB1 mRNA in cells transfected with miR-US5-2 or with GAB1 small interfering RNA (siRNA) or shRNA indicated that expression of GAB1 siRNA or shRNA significantly reduced GAB1 mRNA transcript levels by 4-fold to 5-fold whereas expression of miR-US5-2 had a lesser effect (1.5-fold-to-2-fold reduction) on GAB1 transcript levels (Fig. 1F). These data indicate that miR-US5-2 reduction of GAB1 protein expression levels is primarily mediated through inhibition of protein expression.

### miR-US5-2 alters EGF-mediated MEK/ERK and PI3K signaling pathways.

**FIG 2:**
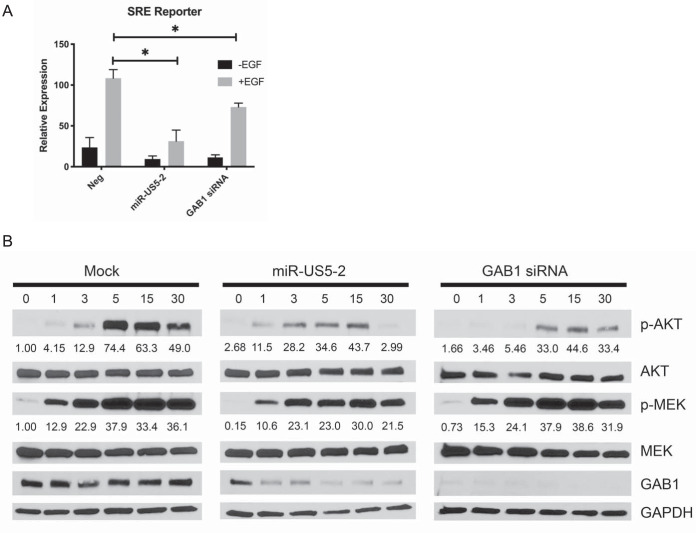
HCMV miR-US5-2 and downregulation of GAB1 attenuate MEK/ERK and PI3K signaling. (A) HEK293T cells were transfected with an SRE luciferase reporter construct along with negative-control miRNA, miR-US5-2 mimic, or a GAB1 siRNA. After 24 h, cells were serum starved for 4 h and treated with EGF (5 ng/ml) for an additional 4 h or left untreated. Cells were lysed, and luciferase expression was measured. Experiments were performed in triplicate, and data are presented relative to results from negative-control transfected cells treated with EGF. Data are presented as standard errors of the means. *, *P* < 0.05 (as determined by two-tailed two-sample *t* test). (B) NHDF were transfected with negative-control miRNA, miR-US5-2 mimic, or a GAB1 siRNA. After 48 h, cells were serum starved for 4 h and then stimulated with EGF (0.05 nM). Protein lysates were harvested at the indicated times and subjected to immunoblotting for phosphorylated and total MEK and AKT as well as GAB1 and GAPDH. Band intensity of p-AKT and p-MEK samples was measured using ImageJ software, and results are presented as a ratio of the band intensity of p-AKT or p-MEK to total AKT and total MEK, respectively. The ratio for the Mock time = 0 time point was set to a value of 1, and each subsequent ratio is presented as a multiplier of the reference sample.

Since GAB1 is a key EGFR adaptor protein that is required for amplification of EGF-mediated MEK/ERK signaling (37), we assessed the effects of miR-US5-2 on a reporter containing the minimal ERK-dependent serum response element (SRE) driving luciferase expression. HEK293T cells were transfected with the reporter as well as negative-control miRNA, miR-US5-2 mimic, or a GAB1 siRNA. After serum starvation, cells were treated with EGF and analyzed for luciferase expression. As shown in Fig. 2A, expression of miR-US5-2 and the GAB1 siRNA significantly reduced EGF-mediated SRE-driven luciferase expression compared to the levels seen with negative-control transfected cells.

GAB1 is the only known adaptor protein linking the EGFR with the PI3K signaling pathway (39–41). To assess the effect of miR-US5-2 on PI3K as well as to confirm its role in modulating MEK/ERK signaling, NHDF were transfected with negative-control miRNA, miR-US5-2 mimic, or GAB1 siRNA. At 48 h posttransfection, the cells were serum starved and treated with EGF for 1 to 30 min, and phosphorylation of AKT and MEK was analyzed by Western blotting. As shown in Fig. 2B, expression of miR-US5-2 and a GAB1 siRNA delayed and altered the kinetics of AKT and MEK phosphorylation in response to EGF stimulation compared to negative-control transfected cells. Expression of both miR-US5-2 and the GAB1 siRNA diminished the phosphorylation of AKT by half in response to EGF at 15 min poststimulation and up to 6-fold at 30 min poststimulation in miR-US5-2 transfected cells. As well, miR-US5-2 and the GAB1 siRNA prevented sustained MEK phosphorylation as evidenced by a decrease in levels of phosphorylated protein at 30 min posttransfection, as was previously described for knockdown of GAB1 (37). Thus, these data suggest that, like knockdown of GAB1 expression by siRNA, miR-US5-2 has the ability to block PI3K and MEK/ERK signaling in response to EGF.

### miR-US5-2 targeting of GAB1 results in a block to cellular proliferation.

**FIG 3:**
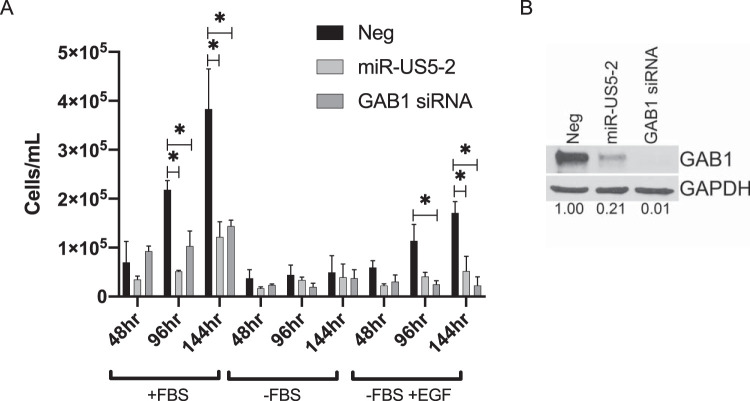
HCMV miR-US5-2 and downregulation of GAB1 affect proliferation of human fibroblasts. (A) NHDF were transfected with negative-control miRNA, miR-US5-2 mimic, or a GAB1 siRNA. 48 h later, cells were trypsinized, counted, and replated at a density of 5,000 cells/well in a 24-well plate in complete media (+FBS), in media lacking FBS (-FBS), or in media lacking FBS but with the inclusion of exogenous EGF (5 ng/ml) (-FBS +EGF). After an additional 48, 96, or 144 h, total viable cells were counted (*n* = 2). *, *P* < 0.05 (as determined by two-tailed two-sample *t* test). (B) At 48 h posttransfection, 2 × 10^5^ cells from the experiment performed as described for panel A were lysed and subjected to immunoblotting for GAB1 and GAPDH. Band intensity was calculated using ImageJ software. The ratio of GAB1 to GAPDH band intensities was set to 1 for the Neg sample, and each subsequent sample ratio is presented as a multiplier of the Neg time point.

As both PI3K signaling and MEK/ERK signaling are important for cell survival and proliferation, we tested whether expression of miR-US5-2 or a GAB1 siRNA would alter the growth of human fibroblasts. NHDF were transfected with miR-US5-2 mimic, GAB1 siRNA, or a negative-control miRNA. At 48 h posttransfection, cells were counted, normalized, and replated followed by total cell counts performed 2, 4, and 6 days later. As shown in Fig. 3A, both miR-US5-2 and the GAB1 siRNA inhibited proliferation of NHDF in complete media (with fetal bovine serum [+FBS]) at all indicated time points. To test whether miR-US5-2 and a GAB1 siRNA were specifically capable of preventing EGF-mediated cellular proliferation, a portion of cells were plated and then the media were changed to either serum-free media (without EGF supplementation) (-FBS) or serum-free media supplemented with EGF (-FBS +EGF) after 2 h. The data shown in Fig. 3A demonstrate that miR-US5-2 and a GAB1 siRNA also blocked cell proliferation specifically mediated by EGF, while the data in Fig. 3B show the effectiveness of miRNA and siRNA knockdown of GAB1 protein levels at the time that the cells were replated. These data indicate that miR-US5-2 is an important regulator of EGFR-mediated signaling to reduce cellular proliferation.

### miR-US5-2 targeting of GAB1 regulates UL138 protein expression.

**FIG 4:**
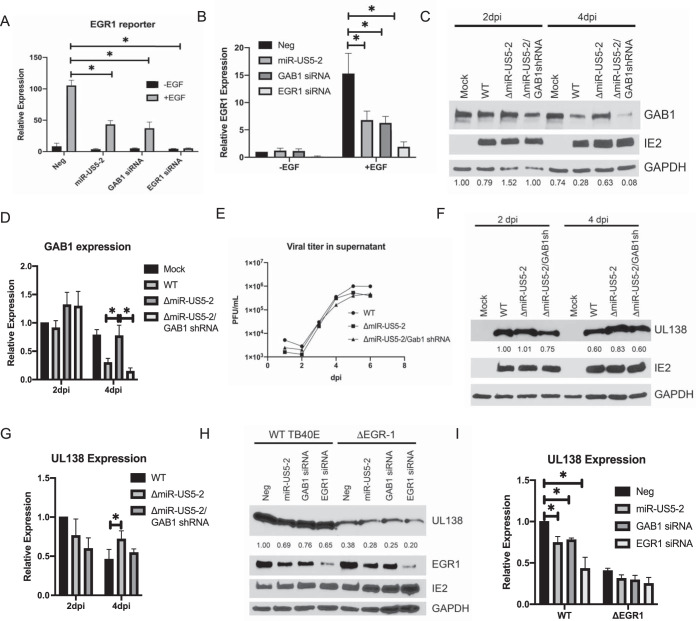
HCMV miR-US5-2 affects UL138 expression during viral infection through targeting GAB1 for downregulation. (A) HEK293T cells were transfected with an EGR-1 luciferase reporter construct along with negative-control miRNA, miR-US5-2 mimic, and GAB1 or EGR-1 siRNA. After 24 h, cells were serum starved for 4 h and treated with EGF (5 ng/ml) for an additional 4 h or left untreated. Cells were lysed, and luciferase expression was measured. Experiments were performed in triplicate, and data are presented relative to results from negative-control transfected cells treated with EGF. Data are presented as standard errors of the means. *, *P* < 0.05 (as determined by two-tailed two-sample *t* test). (B) NHDF were transfected and treated as described for panel A, with EGF (5 ng/ml) being added for 1 h. RNA was harvested, and qRT-PCR was used to analyze EGR-1 expression normalized to 18S. *, *P* < 0.05 (as determined by two-tailed two-sample *t* test). (C) NHDF were infected with the indicated viruses at a multiplicity of infection (MOI) of 3 for 2 and 4 days, after which time protein lysates were harvested and subjected to immunoblotting for GAB1, HCMV IE2, and GAPDH. Band intensities were calculated using ImageJ software. The ratio of GAB1 to GAPDH band intensities was set to 1 for the Mock 2 day postinfection (dpi) sample, and each subsequent sample ratio is presented as a multiplier of the Mock time point. The experiment was performed in duplicate. (D) Quantitation of GAB1 protein levels compared to GAPDH from 2 Western blots using ImageJ software as described for panel C. (E) NHDF were infected as described for panel C, cell supernatants were harvested at the indicated time points, and titers were determined on NHDF. (F) NHDF were infected as described for panel C, and protein lysates were harvested at the indicated times and subjected to immunoblotting for HCMV UL138, IE2, and GAPDH. Band intensities were calculated using ImageJ software. The ratio of UL138 to GAPDH band intensities was set to 1 for the WT 2 dpi sample, and each subsequent ratio is presented as a multiplier of the WT 2 dpi time point. The experiment was performed in triplicate. (G) Quantitation of UL138 expression compared to GAPDH from 3 Western blots using ImageJ software as described for panel D. (H) NHDF were transfected with negative-control miRNA, miR-US5-2 mimic, or siRNAs targeting GAB1 or EGR1 followed by infection with WT or ΔEGR1 viruses. At 48 h later, infected cells were serum starved and treated with EGF (5 ng/ml) for 2 h, after which time protein lysates were obtained and immunoblotted for UL138, IE2, and GAPDH. Band intensities were calculated using ImageJ software. The ratio of UL138 to GAPDH band intensities was set to 1 for negative-control transfected WT samples, and each subsequent ratio was presented as a multiplier of the negative-control transfected WT sample. Experiment was performed in duplicate. (I) Quantitation of UL138 expression compared to GAPDH from 2 Western blots using ImageJ software as described for panel G.

We next investigated whether miR-US5-2 affects additional downstream events mediated by EGF binding to the EGFR by assessing the effect of miR-US5-2 and a GAB1 siRNA on an EGR1-driven luciferase reporter assay. EGR1 is an immediate early transcription factor whose expression is also induced by MEK/ERK-dependent activation of the Elk-1 transcription factor (42, 43). As shown in Fig. 4A, expression of miR-US5-2 or siRNAs targeting GAB1 or EGR1 resulted in reduced EGR1 reporter activity in response to EGF. Additionally, a reduction in the levels of EGR1 transcripts following EGF stimulation in cells transfected with miR-US5-2 or siRNAs targeting GAB1 or EGR1 is shown in Fig. 4B, confirming that miR-US5-2 targeting of GAB1 acts to reduce EGR1 expression in response to EGF.

The expression of HCMV UL138 is regulated, at least in part, through EGR1 binding sites upstream of the UL138 transcriptional start site (14). We hypothesized that miR-US5-2, through modulating the MEK/ERK signaling pathway and EGR1 expression, regulates UL138 expression in response to EGF. To assess the role of miR-US5-2 targeting of GAB1 in UL138 expression in the context of viral infection, we generated a miR-US5-2 knockout virus (ΔmiR-US5-2; 8) and a virus where miR-US5-2 was replaced with an shRNA targeting GAB1 (as identified in Fig. 1E) (ΔmiR-US5-2/GAB1shRNA). As shown in Fig. 4C, GAB1 protein levels were elevated in cells infected with a virus lacking miR-US5-2 compared to cells infected with WT virus, indicating that GAB1 is a target of HCMV during viral infection. By expressing a GAB1 shRNA from the miR-US5-2 region, the levels of GAB1 were reduced compared to those seen with the parental ΔmiR-US5-2 virus and were similar to those measured for the WT infection. Quantitation of multiple blots is shown in Fig. 4D. Single-step growth curve analysis indicates that the mutant viruses replicated with kinetics similar to WT virus kinetics (Fig. 4E). We next analyzed UL138 expression at day 2 and day 4 postinfection of human fibroblasts infected with WT, ΔmiR-US5-2, and ΔmiR-US5-2/GAB1shRNA viruses. As shown in Fig. 4F, at 4 days postinfection, UL138 expression was increased in cells infected with the ΔmiR-US5-2 mutant virus, but was reduced in cells infected with the ΔmiR-US5-2/GAB1shRNA virus, indicating that through targeting GAB1, miR-US5-2 modulates UL138 expression during viral infection. Quantitation of multiple blots is shown in Fig. 4G. We have previously shown that miR-US5-2 also targets the EGR1 transcriptional repressor NAB1 (8). In order to determine if miR-US5-2 affects UL138 expression through modulating NAB1, we infected fibroblasts with a miR-US5-2 mutant expressing an shRNA targeting NAB1 (8). As shown in Fig. S1 in the supplemental material, expression of a NAB1 shRNA in the context of HCMV infection does not significantly affect UL138 expression compared to the parental ΔmiR-US5-2 virus, indicating that miR-US5-2 targeting of GAB1, and not NAB1, mediates the effect of the miRNA on UL138 protein levels. Finally, in order to demonstrate that miR-US5-2 regulation of UL138 protein expression operates through modulation of EGR1 levels during infection, we transfected human fibroblasts with negative-control miRNA, miR-US5-2 mimic, or siRNAs targeting GAB1 or EGR1 followed by infection with WT virus or a ΔEGR-1 mutant virus where an EGR1 binding site upstream of the UL138 transcriptional start site, previously shown to regulate UL138 expression in response to EGF, was mutated (14). Overexpression of miR-US5-2 or expression of siRNAs targeting GAB1 or EGR1 was capable of decreasing UL138 expression levels in cells infected with WT virus, as has been previously reported for EGR1 siRNA (14), but residual UL138 expression present in cells infected with the ΔEGR-1 virus was affected to a lesser extent (Fig. 4H). Quantitation of multiple blots is shown in Fig. 4I. These data indicate that the ability of miR-US5-2 to regulate UL138 expression during infection operates at least partially through attenuating EGFR signaling and downstream EGR1 expression.

10.1128/mSphere.00582-20.1 FIG S1 miR-US5-2 targeting of NAB1 does not affect UL138 expression. (A) NHDF were infected with the indicated viruses at an MOI of 3 for 96 h after which time protein lysates were harvested and subjected to immunoblotting for UL138, IE2, and GAPDH. UL138 band intensity was calculated using ImageJ and compared to the intensity of GAPDH. The ratio of the band intensities of UL138 and GAPDH was set to 1 for the WT sample, and each subsequent ratio is presented as a multiplier of the value set for the WT sample. (B) Quantitation of UL138 band intensity over 4 Western blots. *, *P* < 0.05 (as determined by two-tailed two-sample *t* test). Download FIG S1, EPS file, 1.6 MB. Copyright © 2020 Hancock et al. 2020 Hancock et al. This content is distributed under the terms of the Creative Commons Attribution 4.0 International license.

## DISCUSSION

**FIG 5:**
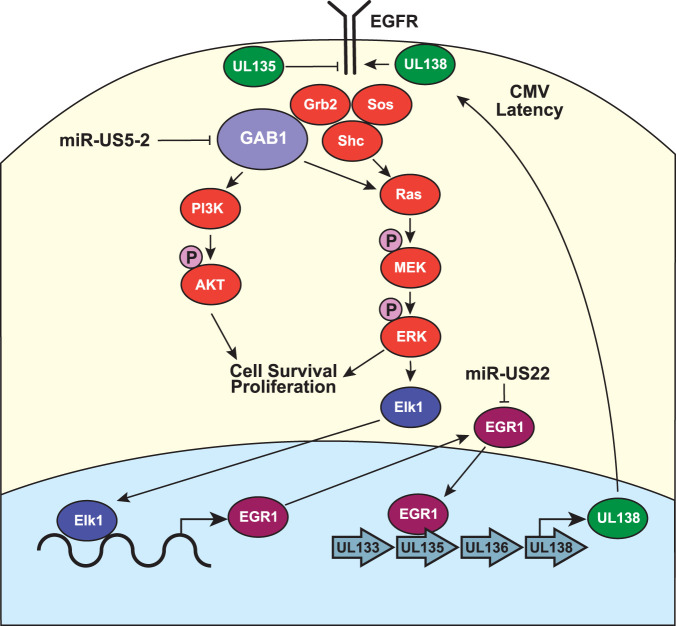
HCMV regulation of EGFR signaling pathways. A proposed model for the interactions of HCMV proteins and miRNAs with the EGFR signaling pathways is presented. Our data demonstrate that miR-US5-2 downregulation of GAB1 dampens MEK/ERK and PI3K signaling pathways, which ultimately affects the expression of EGR1 and UL138. We propose that interference with the EGFR-EGR1-UL138 signaling loop is a component of the switch from latent to lytic replication.

In this report, we show that HCMV miR-US5-2 is capable of attenuating EGF-mediated MEK/ERK and PI3K signaling by targeting the EGFR adaptor protein GAB1 for downregulation. By dampening these key cellular signaling pathways, miR-US5-2 is able to reduce cellular proliferation and regulate expression of downstream transcription factors, including EGR1, which ultimately affects expression of *UL138* (Fig. 5). These results have implications for HCMV latency in CD34^+^ HPCs, where miR-US5-2 regulation of EGR1 could play a role in reducing UL138 expression.

It has previously been shown that regulation of GAB1 levels and activity is critical for modulating the mitogenic and survival pathways activated by EGFR (44, 45), especially at physiological concentrations of EGF (37). The GAB1-p85 interaction is initiated upon GAB1 tyrosine phosphorylation at the EGF receptor. Subsequent activation of PI3K generates 3-phosphoinositide lipids such as PIP_3_, which are bound by the PH domain of GAB1 and anchor the protein near EGFR, resulting in sustained signaling (44, 45). As the EGFR lacks its own p85 binding sites, GAB1 recruitment to the receptor is essential for PI3K activation (41). Additionally, much of the MEK/ERK signaling induced by GAB1 is due to GAB1 sequestration at the plasma membrane by PIP_3_ binding (39), suggesting that the MEK/ERK and PI3K signaling pathways are intimately interconnected. When GAB1 can no longer interact with p85 or SHP2, either through reductions in total or phosphorylated protein levels or by mutations that prevent complex formation, significant reduction in PI3K signaling is observed whereas MEK/ERK signal amplification is lost (37, 39–41, 46, 47). Here, we show that miR-US5-2 phenocopies the knockdown of GAB1 in its modulatory effects on PI3K and MEK/ERK signaling. Thus, by simply fine-tuning GAB1 protein levels during HCMV infection, miR-US5-2 can significantly modulate signal transduction through multiple cellular signaling pathways. Intriguingly, the data presented here suggest that expression of miR-US5-2 may have a more significant effect on MEK/ERK signaling than knocking down GAB1 levels with an siRNA, despite the less efficient knockdown of GAB1 protein (Fig. 1B to D; see also Fig. 2B). This suggests that miR-US5-2 may target additional, currently unknown components of the MEK/ERK signaling pathway that contribute to the overall inhibition of signal transduction.

Given the extensive coevolution between herpesviruses and their hosts and the disparate viral life cycles in different cell types, it is not surprising that regulation of PI3K and MEK/ERK cellular signaling pathways is a hallmark of herpesvirus lytic and latent infection. Like HCMV, herpes simplex virus (HSV) utilizes PI3K and mitogen-activated protein kinase (MAPK) signaling to maintain latency in sensory neurons (48), and inhibition of PI3K results in HSV-1 reactivation (49). HSV VP11/12 stimulates signaling through PI3K (50, 51), while US3-mediated blocking of ERK signaling is important for lytic viral replication (52). During gammaherpesvirus latency, Epstein-Barr virus (EBV) latent membrane protein-1 (LMP-1) acts to maintain EGFR protein levels and downstream ERK and PI3K signaling (53–55) whereas LMP2A directly activates PI3K signaling (56, 57). Additionally, EBV miR-BART7-3p and miR-BART1 directly target PTEN for downregulation, which results in increased AKT activation (58, 59). PI3K signaling is also promoted during Kaposi’s sarcoma-associated herpesvirus (KSHV) latency (60, 61), and inhibition of PI3K signaling stimulates lytic KSHV gene expression (62). Chemical inhibitors of the MEK/ERK and PI3K signaling pathways enhance HCMV reactivation in CD34^+^ HPCs (14), further highlighting the central importance of these signaling pathways in herpesvirus latency. While HCMV downregulation of GAB1 protein results in dampened PI3K and MEK/ERK signaling, other viruses utilize the adaptor protein to aid in viral replication. Hepatitis C virus NS5A forms a complex with GAB1 that enhances prosurvival PI3K/AKT signaling to aid in viral persistence (63). Polyomavirus middle T antigen forms an ShcA-Grb2-GAB1 complex in order to activate PI3K signaling for endothelial cell transformation (64). Additionally, coxsackievirus type B3 cleaves GAB1 protein into two fragments utilizing the viral proteinase 2A. The N-terminal fragment of GAB1 plays an important role in increasing infectivity by enhancing ERK1/2 activity through an unknown mechanism (65).

Mitogenic and prosurvival signaling pathways downstream of EGF binding to the EGFR are key to mediating cell proliferation, and activating mutations in EGFR are commonly found in many types of cancers. EGFR signaling leads to the transcriptional activation of *CYCLIN D1* (66), which helps drive the cell through the G_1_ restriction point. ERK-dependent AP-1 activation transcriptionally upregulates *CYCLIN D1* (67), while AKT phosphorylates and inactivates FOXO1 (68), a *CYCLIN D1* transcriptional inhibitor (69). In addition, both PI3K/AKT signaling and MEK/ERK signaling result in the phosphorylation and degradation of cyclin-dependent kinase inhibitors such as p21 and p27, which help to drive the cells through S phase (70–75). It has previously been shown both *in silico* and *in vitro* that GAB1 is essential for enhancing prosurvival PI3K signaling and extending the duration of mitogenic MEK/ERK signaling, thus playing a critical role in cell proliferation (37). Elevated expression of GAB1 has been observed in human cancers (76), and downregulation of GAB1 by cellular miRNAs has been shown to reduce proliferation in normal and transformed cell types (77–83). Here, we show that miR-US5-2 as well as a GAB1 siRNA can block proliferation of human fibroblasts both under normal growth conditions and specifically in response to EGF. HCMV utilizes multiple means to regulate the cell cycle during lytic infection in order to optimize production of progeny virus (84). Optimal viral gene expression and DNA replication occur in the G_0_ and G_1_ phases, and transit through the G_1_ restriction point and initiation of cellular DNA replication in S phase are blocked (84). Expression of miR-US5-2 and targeting of GAB1 may represent means to limit the extracellular signaling that promotes entry into the cell cycle during lytic and latent infection. Differentiation of HPCs along the myeloid lineage is a key aspect of HCMV reactivation, and limiting cellular proliferation via miR-US5-2-mediated reduction in mitogenic signaling is likely an important factor in promoting differentiation and the reactivation process. miR-US5-2 has recently been shown to block the proliferation of CD34^+^ HPCs under growth-promoting conditions through enhancing the expression of the myelosuppressive cytokine transforming growth factor β (TGF-β) (8). Here, we measured proliferation in serum-free media supplemented only with EGF, indicating that miR-US5-2 blocks proliferation of different cell types by the use of multiple mechanisms.

Downstream of MEK/ERK signaling, EGR1 is an immediate early transcription factor that plays a key role in maintaining the “stemness” of CD34^+^ HPCs (85). EGR1 is highly expressed in CD34^+^ HPCs (85), is transcriptionally induced by EGF treatment (see Fig. 4B), and binds to specific regions upstream of the *UL138* transcriptional start site in order to induce UL138 expression (14). Buehler et al. have shown that UL138 acts to maintain cell surface levels of EGFR (16), setting up a feed-forward loop of EGF-mediated EGR1 expression leading to increased UL138 expression and maintenance of EGFR surface levels, which are critical to maintain latent infection. Intriguingly, HCMV encodes multiple mechanisms to interfere with the EGFR-UL138 feed-forward loop which are important in regulating the switch to viral replication during differentiation and reactivation. HCMV UL135 acts in opposition to UL138 to reduce EGFR surface levels (86), while HCMV miR-US22 directly downregulates EGR1 and affects expression of UL138 (5). Neither UL135 nor miR-US22 is expressed during latent infection of CD34^+^ HPCs (5, 19), but each is induced during the reactivation process. These data suggest that modulation of EGFR signaling is a critical switch to regulate HCMV latency and replication. Here, we show that a second HCMV miRNA, miR-US5-2, blocks EGF-mediated MEK/ERK signaling and induction of EGR1, which also directly affects UL138 protein expression. These data suggest that expression of miR-US5-2 during reactivation, like miR-US22, plays an important role in modulating UL138 levels through reducing GAB1 protein levels and acts in conjunction with UL135 to dampen EGFR-mediated signaling. Unlike miR-US22, miR-US5-2 is detected throughout latency, although expression levels are low compared to those seen with other HCMV miRNAs (4–6). The detection of miR-US5-2 during latent infection suggests that the miRNA may modulate the EGFR signaling stimulated by UL138, perhaps as a mechanism to fine-tune the responses induced by exogenous EGF. Unfortunately, we have been unable to recover miR-US5-2 mutant virus from latently infected CD34^+^ HPCs; thus, the importance of miR-US5-2 regulation of downstream EGFR signaling pathways during latency and reactivation in CD34^+^ HPCs remains to be directly investigated. These data suggest that although miR-US5-2 is not required for replication in fibroblasts, it is essential for latency in CD34^+^ HPC.

Additional roles for miR-US5-2 during HCMV infection have been described, including modulation of TGF-β expression through targeting the transcriptional repressor NAB1 (8), mediation of viral assembly compartment formation through targeting components of the endocytic recycling pathway (87), and inhibition of apoptosis through downregulation of Fas (38). Here, the results associated with the use of a viral mutant in which miR-US5-2 was replaced by an shRNA targeting GAB1 indicate that the ability of the miRNA to modulate UL138 protein levels is mediated specifically through downregulation of the GAB1 protein and modulation of MEK/ERK signaling. Although NAB1 is also a target of miR-US5-2 (8) and is a transcriptional repressor of EGR1 (88), our data demonstrate that NAB1 is not involved in regulating UL138 expression during infection (see Fig. S1 in the supplemental material). HCMV encodes multiple mechanisms to carefully modulate EGFR signaling pathways during lytic infection as well as during latency and must abrogate this signaling in order to reactivate viral gene expression and enable productive progeny virus production. The use of viral miRNAs represents an efficient means to modulate signaling pathways during latency given their abundance, nonimmunogenic nature, and capacity to target multiple cellular and viral proteins (20). The data presented here underscore the important role that HCMV miRNAs play in modulating cell signaling pathways not just to alter the cellular environment but also to control viral protein expression.

## MATERIALS AND METHODS

### Cells, virus, and reagents.

Normal human dermal fibroblasts (NHDF), human aortic endothelial cells (hAECs), and 293T cells were obtained from the American Type Culture Collection. NHDF and 293T cells were maintained in Dulbecco’s modified Eagle’s medium (DMEM) supplemented with 5% fetal bovine serum (FBS; HyClone), 100 U/ml of penicillin, and 100 μg/ml of streptomycin (Invitrogen). hAECs were maintained in EGM-2 media (Lonza) with associated supplements (excluding heparin), as well as 10% FBS, penicillin, and streptomycin.

Viruses used in this study included bacterial artificial genome (BAC)-generated WT TB40/E virus expressing green fluorescent protein (GFP) from the simian virus 40 (SV40) promoter (89) and a TB40/E mutant virus lacking the pre-miR-US5-2 sequence generated by *galK*-mediated recombination (8). Additionally, a mutant was created by *galK*-mediated recombination where the miR-US5-2 region was replaced with a GAB1 shRNA cassette (TGCTGTTGACAGTGAGCGAGTTAACACACTCGTAGTATTTAGTGAAGCCACAGATGTAAATACTACGAGTGTGTTAACATGCCTACTGCCTCGGA) which had been previously cloned into the pLCE expression plasmid using miREXhoFwd (TGAACTCGAGAAGGTATATTGCTGTTGACAGT) and miREOligoRev (TCTCGAATTCTAGCCCCTTGAAGTCCGAGGCAGTAGGC). This shRNA sequence was also subcloned into a pSIREN expression vector (Clontech). The sequences of primers used for amplification of the GAB1 shRNA construct in place of the miR-US5-2 sequence were as follows: for miR-US5-2 shRNA F, CGAGAGCGTTCATCGGGGCATGAAGTACGCATTACACAAACTCCATATATTTGTTACGATAGAATACGGAACGGAGGTATATTGCTGTTGACAGTGAGCG; for miR-US5-2 shRNA R, TATGCACAAAAGGTATGTGTGAATGGAAATACATGATGAATGTCATCATCACGCAAAGCAGCCGTGGGAATGGTGAAGTCCGAGGCAGTAGGCA.

The primers used for cloning miR-US5-2 into the pSIREN expression vectors were as follows: miR-US5-2 F (TGACGAGAGCGTTCATCGG) and miR-US5-2 R (CCGTATGCACAAAAGGTATGTG). NHDF were infected with HCMV at 3 PFU/cell and hAECs with HCMV at 5 PFU/cell for 2 h at 37°C. After that, the inoculum was removed and replaced with fresh medium and samples were harvested as appropriate for each experiment.

Recombinant human EGF was obtained from R&D Systems. siRNAs targeting GAB1 and EGR1 were obtained from Thermo Fisher Scientific.

### Quantitative real-time PCR (qRT-PCR).

Total RNA was isolated from transfected or infected cells using the TRIzol RNA isolation method. cDNA was prepared using 1,000 ng of total RNA and random hexamer primers. Samples were incubated at 16°C for 30 min, 42°C for 30 min, and 85°C for 5 min. Real-time PCR (TaqMan) was used to analyze cDNA levels in transfected or infected samples. An ABI StepOnePlus real-time PCR machine was used with the following program for 40 cycles: 95°C for 15 s and 60°C for 1 min. GAB1, EGR1, and 18S primer/probe sets were obtained from Thermo Fisher Scientific. Relative expression levels were determined using the threshold cycle (ΔΔ*C_T_*) method with 18S as the standard control.

### Immunoblotting.

Protein extracts were run on an 8% SDS-PAGE gel, transferred to Immobilon-P transfer membranes (Millipore Corp., Bedford, MA), and visualized with antibodies (Abs) specific for GAB1 (Cell Signaling), p-MEK (Ser217/221; Cell Signaling), total MEK (Cell Signaling), p-AKT (Thr 308; Cell Signaling), total AKT (Cell Signaling), IE86 (monoclonal antibody [MAb] 810; Millipore), UL138 (14), and GAPDH (glyceraldehyde-3-phosphate dehydrogenase) (Abcam). Relative levels of intensity of bands detected by Western blotting were quantitated using ImageJ software. In each case, the relative intensity of each band was compared to that of the control protein (total MEK or total AKT [Fig. 2] or GAPDH [Fig. 1, 3, and 4]). The ratio of sample to control was set to a value of 1 for the mock treatment (Mock) or the first time point, and each subsequent sample/control ratio is presented as a multiplier of the reference time point.

### Luciferase 3′ UTR assay.

The putative 3′ UTR of GAB1 was cloned into the Dual-Luciferase reporter pSiCheck2 (Clontech) using the following primers: GAB1 F (GATTGAGTTTGGTGTGCAAGC) and GAB1 R (CCATGGCTTCTCATAGTTCAG). Site-directed mutagenesis was performed using the QuikChange PCR method to mutate the potential miR-US5-2 site within the GAB1 3′ UTR. The primers used for site directed mutagenesis were as follows: GAB1 SDM F (CTGTAGATACTGTTCTTGGGTGTTC) and GAB1 SDM R (GAACACCCAAGAACAGTATCTACAG). 293T cells seeded into 96-well plates were cotransfected in triplicate with 100 ng of plasmid and 100 fmol of miRNA mimic (custom designed; IDT) using Lipofectamine 2000 (Invitrogen). Cells were incubated overnight and then harvested for luciferase assay using a Dual-Glo reporter assay kit (Promega) according to the manufacturer’s protocol.

### Luciferase reporter assays.

For SRE and EGR1 reporter assays, 293T cells were plated as described above and transfected with a 100-ng volume of the SRE or EGR1 Cignal reporter plasmid (Qiagen); 25 ng of pRLSV40-Rluc; and 100 fmol of miRNA mimic, GAB1, or EGR1 siRNA using Lipofectamine 2000. Cells were incubated overnight, and then the media were replaced the next day with serum-free media. After 4 h of serum starvation, 100 pg/ml EGF was added to serum-free media for 4 h and then the cells were harvested and analyzed as described above. Luminescence was detected using a Veritas microplate luminometer (Turner Biosystems). All experiments were performed at least in triplicate, and results are presented as means ± standard deviations.

### Cell proliferation assays.

NHDF seeded in 12-well plates were transfected with 40 μM miR-US5-2 mimic or GAB1 siRNA by the use of RNAiMax (Thermo Fisher Scientific) according to the manufacturer’s instructions. After 48 h, cells were trypsinized, counted, and replated at a density of 5,000 cells/well in duplicate. Total cell numbers were counted at 2, 4, and 6 days after replating using a Countess automated cell counter (Thermo Fisher Scientific).

### Statistical analysis.

Unpaired Student’s *t* tests (Microsoft Excel software) were used to determine *P* values. Results were considered significant at a probability (*P*) value of <0.05.
